# King Edward's Hospital Fund for London

**Published:** 1909-03-20

**Authors:** 


### HOSPITAL ADMINISTRATION.

### CONSTRUCTION AND ECONOMICS.

### KING EDWARD'S HOSPITAL FUND FOR LONDON.

ANNUAL MEETING AT MARLBOROUGH HOUSE.

The annual meeting of the President and General Council of King Edward's Hospital Fund for London, to receive and adopt the accounts and the Report for the year 1908, was held at Marlborough House on the 10th inst., his Royal Highness the Prince of Wales, President of the Fund, being in the chair.

Sir Wm. Church (Chairman of the Distribution Committee) moved, and the Lord Mayor (Sir G. W. Truscott) seconded, a resolution with reference to the payment of an amalgamation grant.

Lord Rothschild, the Hon. Treasurer, in presenting the account of receipts and expenditure and the balance-sheet for the year ending December 31, 1908, called attention to the fact that the income from investments included a bonus dividend of £17,000, on securities given by Lord Mount-Stephen and Lord Strathcona. The balance-sheet now presented was of a rather more complicated nature than usual, because by the Act of Parliament they had to invest in trust funds those moneys left without any particulars being specified, and only in non-trust funds those which the donors desired should be so invested. When the separation of these funds took place, on July 21, the non-trust funds were worth £100,000 more than they cost, and on December 31 there was a further increase of £100,000. At the latter date the trust funds showed an increase of only £1,000. He looked forward, with the advice of the gentlemen with him on the Finance Committee, to the successful administration of the funds entrusted to them, and he hoped they would have many opportunities of acknowledging gifts of money both for trust and non-trust funds.

The Prince of Wales moved the adoption of the accounts, which was seconded by Sir Edgar Speyer, and carried unanimously.

Sir Savile Crossley (Hon. Secretary) presented the draft Report of the Council for the year 1908.

#### The Prince's Speech.

The Prince of Wales, in moving the adoption of the Report, said: My Lords and Gentlemen,—You will, I hope, agree with me in thinking that we may congratulate ourselves upon the condition of the affairs of the Fund as described in the report which lies on the table. I do not propose to go at length into the various questions to which it refers, as the business of the Fund was fully dealt with at our meeting in December. The Act of Incorporation has worked most satisfactorily and smoothly during the past year. It has been necessary to hold a considerable number of committee meetings, but the members have, as usual, responded readily to the calls made upon their time. The completion of the negotiations for the amalgamation of the Hampstead and North-West London Hospitals will, I feel sure, be approved of by the charitable public, both as conducing to the better organisation of hospital accommodation in that neighbourhood, and as one more step in the direction of economy of management. It will be remembered that the Orthopædic Hospitals were the first to combine, and it is satisfactory to know that the new buildings in Great Portland Street will be ready for occupation by the three amalgamated institutions in the course of this year. Last March we looked forward to the appointment of a committee to inquire into the system prevailing in the London voluntary hospitals with regard to the admission of out-patients. The Report of the Poor-law Commission which we were waiting for has, as you know, quite recently appeared, and will require most careful consideration. As it is probable that the whole of the evidence upon which the report is based will not be issued for some considerable time, I have decided, upon the advice of the Executive Committee, to defer the appointment of our committee of inquiry until next year. I have now much pleasure in moving the adoption of the report. (Hear, hear.)

The Bishop of Southwark, in seconding the motion, said it was with real satisfaction that they had listened to what the Chairman of the Finance Committee had been able to report as to the condition of the Fund. If they looked at either end of their support, at the big end, or the little end, the results were equally satisfactory. On the one hand, it was not at all certain that benefactions so great as those of the late Mr. Lewis, and of Lord Mount-Stephen, Lord Strathcona, and Lord Iveagh would have come to the hospitals of London had there not been a Fund like this acting as a focus, accredited by the patronage of his Majesty and of his Royal Highness, and known to be managed as this Fund was. At the other end, it was satisfactory to note that the League of Mercy continued its beautiful work of gathering in the small donations of those who gave with as much feeling and perhaps with as much sacrifice as the wealthier donors.

The resolution was then put and carried unanimously.

In the absence of the Earl of Bessborough (Chairman of the Executive Committee) Mr. Stuart-Wortley presented an interim report of the Executive Committee upon the question of rules and regulations, which was adopted.

Mr. Hugh C. Smith moved, and the Master of Elibank seconded, a vote of thanks to the Prince of Wales for presiding, which was carried by acclamation. His Royal Highness said: My Lords and Gentlemen,—I much appreciate the very kind manner in which the vote of thanks has been proposed and received. The large attendance of members at these meetings, and the hearty support which you invariably give me, are most gratifying, and greatly encourage me in my work as President of the Fund in which I am so deeply interested.

### GRANTS FROM THE KING'S HOSPITAL FUND FOR 1909.

We are asked to state that hospitals in the County of London, or within nine miles of Charing Cross, desiring to participate in the grants made by this Fund for the year 1909 must make application before the 24th inst. to the Hon. Secretaries, 7 Walbrook. Applications will also be considered from convalescent homes and sanatoria for consumption which are situated within the above boundaries, or which, being situated outside, take a large proportion of patients from London.

